# Ethyl acetate subfractions from ethanol extracts of fermented oats (*Avena sativa* L.) exert anti-cancer properties *in vitro* and *in vivo* through G2/M and S Phase arrest and apoptosis

**DOI:** 10.7150/jca.48993

**Published:** 2021-01-30

**Authors:** Nanhai Zhang, Liang Zhao, Shengbao Cai, Xiang Zeng, Wei Wu, Baoping Ji, Feng Zhou

**Affiliations:** 1 Beijing Key Laboratory of Functional Food from Plant Resources, College of Food Science and Nutritional Engineering, China Agricultural University, Beijing 100083, China.; 2 Beijing Engineering and Technology Research Center of Food Additives, Beijing Technology & Business University (BTBU), Beijing 100048, China.; 3 Yunnan Institute of Food Safety, Kunming University of Science and Technology, Kunming 6505000, China.; 4 College of Engineering, China Agricultural University, Beijing 100083, China.

**Keywords:** anti-cancer, oats, fermentation, apoptosis, cell cycle arrest, ROS

## Abstract

**Background:** Cancer is a major public problem and poses a long-term impact on patients' life, work, and study. Oats are widely recognized as healthy food and fermented oats were rich in the higher contents of polyphenols. However, the role of fermented oats in cancer remains elusive.

**Methods:** The effect of ethyl acetate subfractions (EASs) from ethanol extracts of oats fermented by *Rhizopus oryzae* 3.2751 on cancer cells was verified by series experiments *in vitro* and* in vivo*. The cell viability, colony formation, cell cycle, apoptosis, reactive oxygen species (ROS), mitochondrial membrane potential (MMP), and western blot were determined *in vitro*. The toxicity of EASs and xenograft mouse model were performed *in vivo*.

**Results:** MTT assay indicated that EASs interference suppressed the proliferation of four human cancer cells in a dose-dependent manner without a significant impact on two normal cells. EASs (0.2, 0.4, and 0.8 μg/mL) resulted in the G2/M and S phase arrest, apoptosis, depolarization of MMP, and ROS generation in HepG2 cells by flow cytometry. p53, JNK, caspase-9, and caspase-3 were activated and the expression of Bax was promoted, while the expression of Bcl-2 was reduced in HepG2 cells exposed to EASs via western blot. Furthermore, the *in vivo* study using a xenograft mouse model demonstrated that EASs attenuated the tumor growth with low systemic toxicity.

**Conclusions:** EASs exhibited anti-cancer activities *in vitro* and *in vivo* via cell cycle arrest and apoptosis. This finding suggests that polyphenol-enriched composition from fermented oats might become a promising candidate for impeding the development and progression of liver cancer.

## Introduction

Cancer is one of the leading causes of morbidity and mortality worldwide, and its occurrence is expanding because of the aging and established risk factors like smoking, overweight, physical inactivity, and others 1. According to the WHO report, cancer was responsible for 8.8 million deaths in 2015, and nearly 1 in 6 deaths is due to cancer. In China, 2.8 million people died from cancer in 2015 with the growing number of new cases and deaths 2. Although there is a declining tendency for both men and women, as second-leading causes of death in the United States, 609640 cancer deaths are estimated in 2018 3. With the increase of cancer cases, the economic burden from cancer is tremendous and unbearable for patients 4.

At present, chemotherapy and synthetic anti-cancer drugs are the hot topic for cancer treatment. Despite the rapid and notable efficacy of these therapies, they are far from satisfactory results because of adverse effects and resistance in cancer for patients 5. Therefore, it simulated researchers to seek novel and less toxic approaches to protect against cancer, especially natural compounds. Several epidemiological studies supported that the consumption of natural products containing kinds of bioactive components could alleviate the risk of many types of cancer 6, 7. Furthermore, compared to traditional anti-cancer drugs, natural products are regarded as more secure options 8.

As widely recognized healthy food and staple food for humans, whole grain oats become increasingly popular due to its outstanding functional and nutritional composition like phenolic acids, flavonoids, etc., having strong antioxidant capacity 9. Fermented foods are the products made by microbial actions and enzymatic conversions of food components 10. They have been regarded as functional foods with many health benefits 11, 12. Previous reports indicated that not only the contents of total phenolics and flavonoids were higher, but also the γ-aminobutyric acid content increased and phytate content reduced in fungal fermented-oats compared with their counterparts of nonfermented oats, particularly by* Rhizopus oryzae* 3.2751 13. It was further found that subfractions from ethanol extracts of fermented oats using various filamentous fungi could enhance the contents of total phenolics and flavonoids, especially in ethyl acetate subfractions (EASs) bearing responsibility for the strongest antioxidant and inhibitory capacities on pancreatic lipase and oleic acid-induced hepatic steatosis *in vitro* 14, 15. The contents of caffeic, ferulic, chlorogenic, and *p*-coumaric acids, major identified phenolic acids in EASs from native oats notably augmented after fermentation 9. In addition, some fermented fruits, vegetables and grains by microorganisms have been proved to exhibit anti-cancer effects and less toxicity of natural conditions. For example, increases in antioxidant and anti-cancer activities of blueberries, sweet potato, and soy were observed after fermentation by *Lactobacillus plantarum*, *Lactobacillus acidophilus*, and *Bifidobacterium* and *Lactobacillus* Sp., respectively 16-18. Extracts from fermented wheat germ could suppress the growth of various types of cancer cell lines 19. However, whether the fermented whole grain oats have the same anti-cancer activities is still unclear.

Hence, the objective of this study is to evaluate the inhibitory effect of extracts from fermented oats on cancer cells in order to broaden the further utilization of fermented whole grain oats. In the present work, EASs rich in polyphenols were extracted and fractionated from *Rhizopus oryzae* 3.2751-fermented oats. The effect of EASs on cancer cells and its underlying mechanisms of action were investigated* in vitro* and *in vivo*.

## Materials and Methods

### Chemicals and reagents

Dulbecco's modified Eagle's medium (DMEM), RPMI-1640 medium, DMEM/F12 medium, fetal bovine serum (FBS), penicillin and streptomycin were bought from Gibco Life Technologies (Grand Island, NY, USA). Dimethyl sulfoxide (DMSO), propidium iodide (PI), 3-(4,5-dimethylthiazol-2-yl)-2,5-diphenyltetrazolium bromide (MTT), ribonuclease (RNase) A, 2',7'-dichlorofluorescin diacetate (DCFH-DA), crystal violet, and manganese (III) tetrakis-(1-methyl-4-pyridyl) porphyrin (MnTMPyP) were obtained from Sigma-Aldrich (St. Louis, MO, USA). Annexin V-FITC apoptosis detection kit, mitochondrial membrane potential (MMP) assay kit with 5,5',6,6'-tetrachloro-1,1',3,3'-tetraethyl-benzimidazolylcarbocyanine iodide (JC-1), bicinchoninic acid assay kit, RIPA buffer, and phosphate buffered saline (PBS) were purchased from Beyotime Biotechnology Inc. (Beijing, China). Primary antibodies against procaspase-9, cleaved caspase-9, procaspase-3, cleaved caspase-3, cleaved-poly-ADP-ribose polymerase (PARP), Bax, Bcl-2, p-p53, p-c-Jun N-terminal kinase (JNK), and β-actin used were obtained from Santa Cruz Biotechnology (Santa Cruz, CA, USA). Other chemicals and reagents, such as NaCl, ethanol, *n*-hexane, ethyl acetate, etc., were all of analytical grade.

### Microorganism

The *Rhizopus oryzae* 3.2751 applied in this study were obtained from the Institute of Microbiology, Chinese Academy of Sciences (Beijing, China), and maintained in lyophilized form. The fungi were activated in potato dextrose agar and grown at 25 °C for 7 days to produce spores after three passages. The spores from a potato dextrose agar culture were washed and suspended in 20 mL of 0.9% NaCl. The spore suspensions were adjusted to 1.0×10^7^/mL and sustained at 4 °C for further use.

### Preparation of samples

#### Fermentation of oats

Oats (genotype, G4) were purchased from the Chinese Academy of Agricultural Sciences (Beijing, China) and kept at 4 °C for further use. Oats were pretreated and fermented via solid-state fermentation culture with a conidiospore suspension (10^6^ spores/g of oats) for 72 h according to our prevenient prescription 9.

#### Preparation of extracts from fermented oats

The EASs from fermented oats were obtained by the previous protocol with some variations 9. The native and fermented oats were ultrasonically treated at 45 °C for 30 min with a mixture of ethanol-water (80:20) at a volume ratio of 1:5. After centrifugation of cooled extracting solution at 2862 *g* for 15 min, the supernatant was gathered. The extraction steps were repeated once for the residue and the supernatants blended. The mixing solution was then vacuum evaporated at 45 °C to acquire the ethanol extracts. The ethanol extracts were dissolved with 30% aqueous methanol at a volume ratio of 1:10 and extracted with an equal volume of *n*-hexane for three times. The upper solution was removed and the remainder was extracted with an equal volume of ethyl acetate for three times. The upper solution was combined and then evaporated using a rotary evaporator at 40 °C under reduced pressure, vacuum dried to obtain the EASs, and kept in -80 °C for separation by column chromatography.

#### Fractionation of EASs

**Figure 1:**
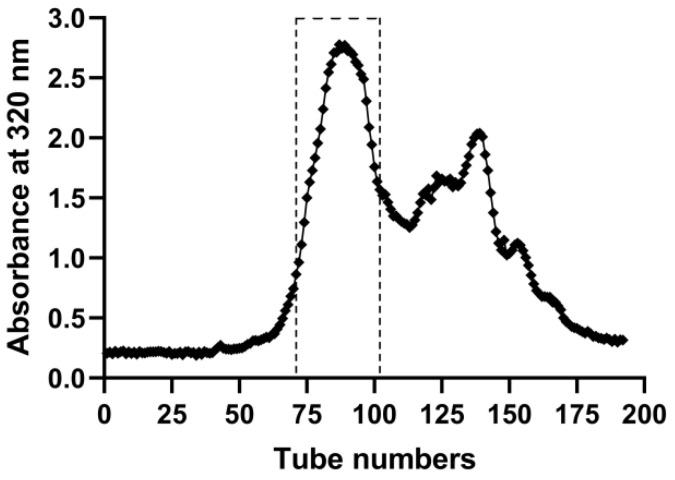
The elution curve of EASs through Sephadex LH-20 column with methanol by detecting absorbance at the wavelength of 320 nm.

For applying to the experiment *in vitro* and *in vivo*, EASs were chromatographed on a Sephadex LH-20 column (40 cm × 2.5 cm i.d., GE Healthcare Bio-Sciences AB, Sweden) to eliminate the part without anti-cancer activities, which was conducted as we previously reported 14. After the column was fulfilled with methanol, 2.0 g EASs dissolved in 1.5 mL methanol was loaded onto the column. Then the column was eluted consecutively with methanol at a flow rate of 6.0 mL/min. The elutes were collected (12 mL/tube) by an automatic fraction collector (DBS-100, Shanghai Hu Xi Analysis Instrument Factory Co. Ltd., Shanghai, China) and its absorbance was determined at 320 nm by SpectraMax M2^e^ microplate reader (Molecular Devices, USA). As shown in Figure 1, the subfractions from 71 to 102 tubes were mixed and then evaporated at 40 °C, followed by lyophilizing the samples by a freeze dryer (LGJ-18, PLA Academy of Military Sciences, Beijing, China).

### *In vitro* studies

#### Cell lines and cell culture

Cell lines were purchased from American Type Culture Collection (ATCC, Manassas, VA, USA). Human cancer cells, including HepG2 (hepatocellular carcinoma), HeLa (cervical carcinoma), and HT-29 (colon carcinoma) cells were cultured in DMEM medium supplemented with 10% FBS, containing 100 units/mL penicillin and 100 μg/mL streptomycin (1% PS). MDA-MB-231 (human breast adenocarcinoma) and 3T3-L1 (mouse preadipocyte) cells were grown in RPMI-1640 medium with 10% FBS and 1% PS. ARPE-19 (human retinal pigment epithelial) cells were cultured in DMEM/F12 medium with 10% FBS and 1% PS. All cell lines were incubated in a humidified incubator at 37 °C in an atmosphere of 5% CO_2_ and 95% air.

#### Cell viability assay

The cytotoxicity of EASs in cells was determined by MTT assay. All cells at a density of 8×10^3^ cells/well were seeded in 96-well plates and incubated for 24 h. Then, the cells were treated with serial concentrations of EASs. After 48 h of culture, the medium was removed and replaced with 150 μL of 0.5 mg/mL MTT. Following the incubation for an additional 4 h, 150 μL of DMSO was added into each well to solubilize the formazan crystals and the absorbance was measured using a microplate reader (SpectraMax M2^e^, Molecular Devices, USA) at 570 nm. Cell viability was expressed as the percentage of absorbance for the treated group with respect to that for the vehicle group which was regarded as 100%. IC_50_ values of EASs on cells were calculated as a concentration suppressing cell viability by 50%.

#### Colony formation assay

Colony formation assay was performed by reference to the procedures of Ding et al. 20. HepG2 cells were plated at 500-700 cells/well on 6-well plates, challenged on EASs at various concentrations, and grew until visible colonies formed for about 14 days. Then, the cells were washed with PBS and stained with 0.5% crystal violet. The number of colonies with at least 50 cells was quantified under a microscope.

#### Cell cycle analysis

For analyzing the cell cycle distribution, flow cytometry was performed as described previously 21. In brief, HepG2 cells (4×10^5^ cells/well) were plated at 6-well plates and challenged on EASs for 24 h and 48 h, respectively. Next, the cells were collected, washed twice with cold PBS, and fixed in 70% cold ethanol at 4 °C overnight. After being washed with chilled PBS, the cells were incubated with RNase A (0.5 mg/mL) at 37 °C for 1 h and then stained with PI (final concentration of 10 μg/mL) at room temperature in the dark for 15 min. Afterward, the cell cycle analysis was carried out on a flow cytometer (FACS Calibur, BD Biosciences, San Jose, USA).

#### Apoptosis detection

HepG2 cells were seeded in 6-well plates at a density of 4×10^5^ cells/well and incubated with EASs for 24 h and 48 h, respectively. After treatment, the cells were harvested and stained with the Annexin V-FITC apoptosis detection kit according to the manufacturer's instructions. Then, stained cells were analyzed by a flow cytometer.

#### Measurement of intracellular reactive oxygen species (ROS)

Intracellular ROS level in treated HepG2 cells was detected using DCFH-DA, a green oxidation-sensitive fluorescent probe following the methods described in Rai et al. with some modifications 22. HepG2 cells were seeded in 6-well plates at 4×10^5^ cells/well and treated with different concentrations of EASs for 6 h. Treated cells were rinsed with PBS, and stained with 1 mL of 25 μM DCFH-DA at 37 °C in the dark for 30 min. After that, the cells were washed twice and re-suspended in PBS buffer. The fluorescence intensity was measured using a flow cytometer.

Furthermore, to confirm whether the production of ROS participates in the apoptosis caused by EASs, MnTMPyP, a superoxide dismutase/catalase mimetic, was used to inhibit the growth of ROS level. HepG2 cells were treated with EASs (0.4 μg/mL) in the presence or absence of MnTMPyP (10 μM, not affecting the cell proliferation) for 48 h. Cell viability and apoptosis were detected by MTT assay and Annexin V-FITC apoptosis detection kit described above.

#### MMP analysis

To assess the disruption of MMP, a lipophilic cationic fluorescent probe JC-1 was utilized. J-aggregates that emitted red fluorescent formed when JC-1 accumulated in the matrix of mitochondrial with high MMP. However, JC-1 existed as a monomer in cells with low MMP, producing green fluorescent. After treatment with EASs for 24 h, HepG2 cells were collected, labeled with the MMP assay kit with JC-1 following the manufacture's recommendations, and analyzed using a flow cytometer.

#### Western blot analysis

After cultivation with EASs for 48 h, proteins from HepG2 cells were extracted in RIPA buffer containing protease inhibitors and phosphatase inhibitors and quantified using a bicinchoninic acid assay kit. Western blot analysis was performed as a previous description using specific primary antibodies for procaspase-9, cleaved caspase-9, procaspase-3, cleaved caspase-3, cleaved-PARP, Bax, Bcl-2, p-p53, p-JNK, and β-actin 23.

### *In vivo* studies

#### Animals

Male and female KM mice (5-6 weeks old) and female BALB/c nude mice (5-6 weeks old) were obtained from Beijing Vital River Laboratory Animal Technology Co., Ltd. (Beijing, China) [Certificate SCXK (Beijing) 2016-0006]. Male and female mice were housed in separated and individual cages with free access to basic feed and water. The mice were kept in an SPF environment under a 12 h light-dark cycle at the room temperature of 18-22 °C and relative humidity of 40-60%. All animals were acclimated for one week before treatment. All animal procedures were executed in compliance with the Animal Ethics Committee of the Beijing Key Laboratory of Functional Food from Plant Resources (Permit number: A330-15) and the guidelines for the care and use of laboratory animals of the National Institutes of Health.

#### Toxicity test of EASs

KM mice were randomly divided into 6 groups with 10 mice each consisting of 5 males and 5 females. Based on the IC_50_ values of inhibiting HepG2 cells proliferation and preliminary studies, EASs were resolved in corn oil and given to mice by intraperitoneal administration at a graded dose of 5, 10, 20, 40, and 60 mg/kg body weight (bw) every day for 24 days. The injection volume was 10 mL/kg bw. The vehicle group was intraperitoneally injected with the same volume of corn oil. Food intake in each group was recorded every 8 days and mice were weighed every 4 days throughout the experiment period. All mice were killed on 24^th^ day after treatment and the main organs (heart, liver, spleen, and kidney) were excised and weighed immediately. Organ weights were normalized to the final body weight as organ indices.

#### Xenograft mouse model

HepG2 cells were collected, counted, and re-suspended in DMEM medium at the concentration of 1×10^6^ cells per 100 μL. Nude mice were inoculated subcutaneously with cell suspensions (0.2 mL per animal) into the right axilla. When the tumor volume approximately grew up to 100 mm^3^, mice with similar tumor volume were assigned randomly into 4 groups (*n* = 7/group), including vehicle group and experimental groups. The mice without evident tumor were removed before grouping. The tumor-bearing mice received an intraperitoneal injection of different doses of EASs (10, 20, and 40 mg/kg bw) or the solvent (corn oil, vehicle group) once a day for 20 days. The dose was chosen according to the toxicological profile of EASs above. The volume of administration was 10 mL/kg bw. During the treatment period, food intake in each group, body weight, and tumor volume of mice were monitored every 4 days. Tumor volume was determined by Vernier caliper and calculated using the following formula: Tumor volume (mm^3^) = (a × b^2^)/2, where a and b are the longest and shortest diameters of tumors, respectively. At the end of the experiment, all mice were weighed and sacrificed. The tumors and spleen tissues were dissected out and weighed. Subsequently, they were fixed with 10% formaldehyde solution, embedded with paraffin, sliced by a microtome (RM2235, Leica Biosystems, Germany), and subjected to hematoxylin and eosin (H&E) staining. The histopathological changes of tissues were observed under a light microscope (DM2000, Leica Microsystems, Germany).

### Statistical analysis

All data were presented as mean ± standard deviation (SD). Three independent experiments were conducted for *in vivo* study. IC_50_ values (μg/mL) from cell viability assay were calculated from dose response curve-fitting via nonlinear regression analysis on GraphPad Prism 8.0 (GraphPad Software, San Diego, CA, USA). Significant differences between experimental and vehicle groups were evaluated with one-way analysis of variance (ANOVA) followed by Tukey's test. Statistical analyses were performed by SPSS 25.0 (SPSS Inc., Chicago, CA, USA). *p <* 0.05 or < 0.01 were considered as statistically or highly statistically significant, respectively.

## Results

### EASs inhibited the growth of cancer cells

**Figure 2:**
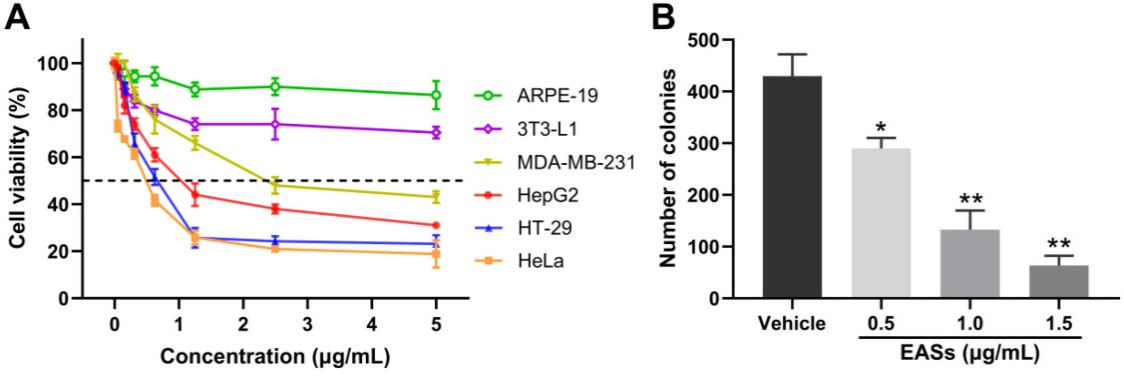
** EASs inhibited the growth of cancer cells.** (A) MDA-MB-231, HepG2, HT-29, HeLa, 3T3-L1, and ARPE-19 cells were treated with increasing concentrations (0.05, 0.156, 0.312, 0.625, 1.25, 2.5, and 5 µg/mL) of EASs for 48 h. Cell viability was measured by MTT assay. (B) HepG2 cells were incubated with different concentrations (0.5, 1, and 1.5 µg/mL) of EASs for 48 h. The number of colonies formed was quantified under a microscope. Data were expressed as mean ± SD (*n* = 3). *p < 0.05, **p < 0.01, vs. vehicle.

To validate whether EASs show anti-cancer activities, we determined the cell viability of four human cancer cells (Figure 2A). As a control, normal cells (3T3-L1 and ARPE-19 cells) were utilized. These cells were exposed to different concentrations of EASs for 48 h. The MTT assay appeared a concentration-dependent cytotoxic effect of EASs on MDA-MB-231, HepG2, HT-29, and HeLa cells, with the IC_50_ values of 2.37, 0.75, 0.63, and 0.54 μg/mL, respectively. However, the normal cells revealed intense resistance on EASs, which demonstrated that EASs selectively suppressed the proliferation of cancer cells, whereas did not pose any apparent cytotoxicity towards normal cells. Liver cancer is common cancer and one of the leading causes of cancer-related deaths worldwide 24. Therefore, we firstly investigated the anti-cancer effect of EASs on HepG2 cells in the subsequent experiment.

The impact of EASs on tumourigenesis of HepG2 cells was estimated by colony formation assays. As depicted in Figure 2B, EASs obviously inhibited the colony formation of HepG2 cells in a dose-dependent manner (*p* < 0.05). Compared with the vehicle group, the number of colonies of HepG2 cells imposed by increasing concentrations (0.5, 1.0, and 1.5 μg/mL) of EASs was decreased by 32.4%, 69.0%, and 85.4%, respectively.

### EASs caused cell cycle arrest in HepG2 cells

**Figure 3:**
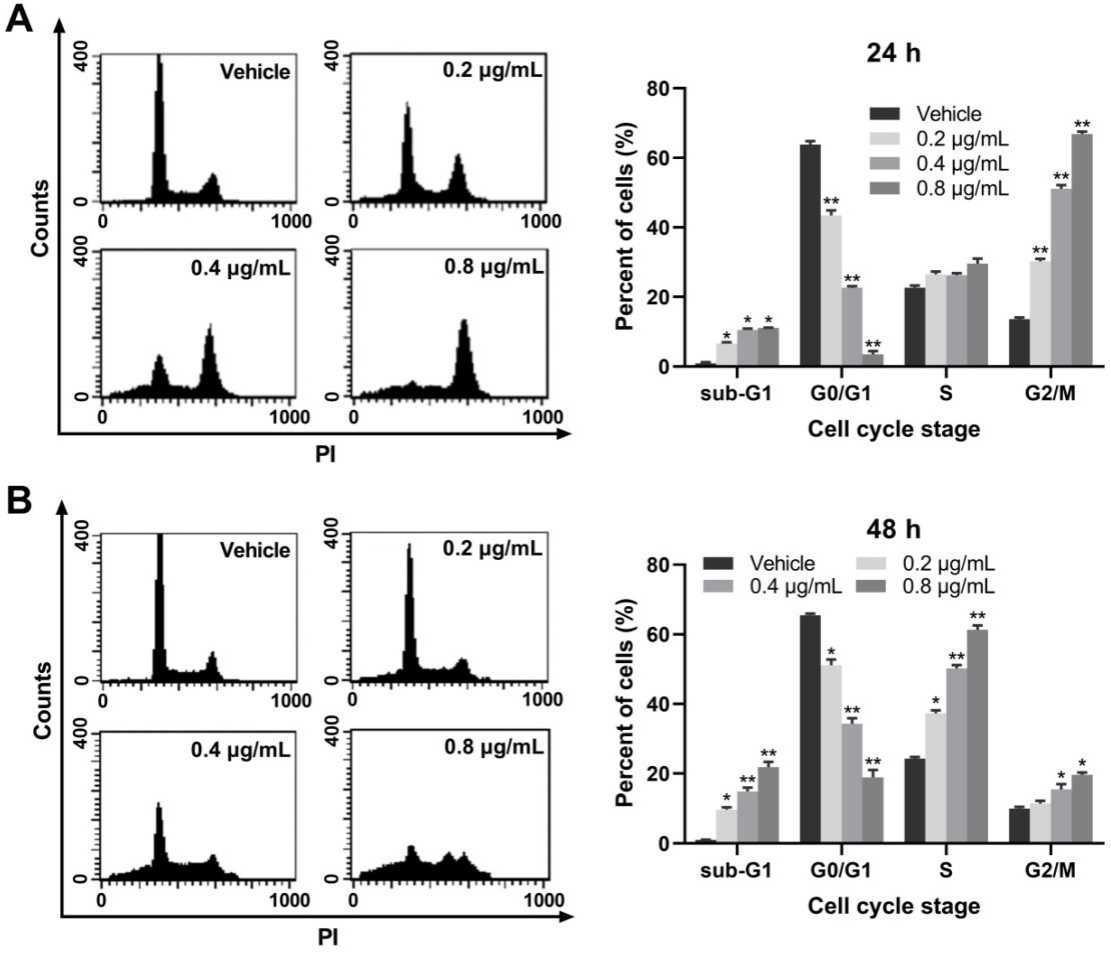
** EASs causes cell cycle arrest in HepG2 cells.** (A and B) Cell cycle distribution in HepG2 cells treated with different concentrations (0.2, 0.4, and 0.8 µg/mL) of EASs for 24 h and 48 h was analyzed on a flow cytometry after staining with PI. Data were expressed as mean ± SD (*n* = 3). *p < 0.05, **p < 0.01, vs. vehicle.

To elucidate the potential mechanisms through which EASs suppressed cell proliferation, the cell cycle progression was detected on a flow cytometry. Our results exerted an evident and dose-dependent influence of EASs on the cell cycle stage in HepG2 cells. As displayed in Figure 3A, after exposed to different doses of EASs (0.2, 0.4, and 0.8 μg/mL) for 24 h, compared to the vehicle group, the results revealed dramatical promotion in the G2/M phase by 2.2-, 3.8-, and 4.9-fold, respectively (*p* < 0.01), accompanied by a prominent decrease in the percentage of cells in the G0/G1 phase from 63.8% in the vehicle group to 43.4%, 22.6%, and 3.5%, respectively (*p* < 0.01). At the same time, HepG2 cells were accumulated marginally in sub-G1 phase (*p* < 0.05) and had no distinct changes in the S phase. Moreover, when incubated with different doses of EASs (0.2, 0.4, and 0.8 μg/mL) for 48 h, a considerable upregulation of 13%, 25.9%, and 37% in the S population of HepG2 cells with respect to 24.3% of that in the vehicle group was observed, respectively (Figure 3B, *p* < 0.05). Similarly, the number of cells in the sub-G1 and G2/M phases augmented (*p* < 0.05), with the same reduction of the cell numbers in G0/G1 phase (*p* < 0.05). Overall, EASs induced G2/M phase arrest at the initial stage of treatment and also produced S phase arrest at the later period of treatment. EASs inhibited the cell growth of HepG2 cells not only through cell cycle arrest, but also through inducing apoptosis due to the significant increase of the number of cells in sub-G1 phase.

### EASs aroused apoptosis in HepG2 cells

**Figure 4:**
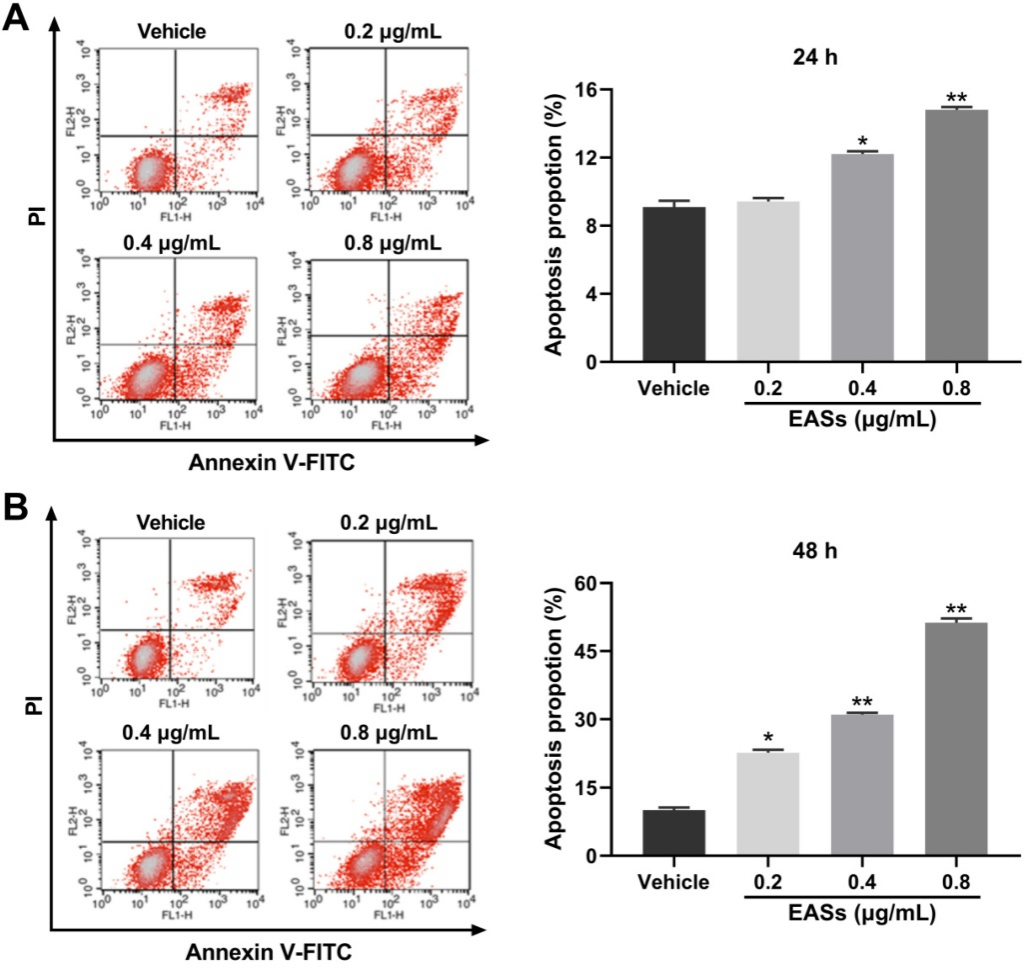
** EASs aroused the apoptosis in HepG2 cells.** (A and B) After treatment with different concentrations (0.2, 0.4, and 0.8 µg/mL) of EASs for 24h and 48 h, HepG2 cells were stained with Annexin V-FITC/PI. Apoptotic rate was detected on a flow cytometry. Data were expressed as mean ± SD (*n* = 3). *p < 0.05, **p < 0.01, vs. vehicle.

Cell apoptosis was further analyzed by flow cytometry using Annexin V-FITC/PI staining. Figure 4 presented that EASs led to apparent apoptosis in HepG2 cells in a concentration-dependent manner for 24 h and 48 h. Specially, the population of apoptotic cells rose from 10% in the vehicle group to 22.6% (*p* < 0.05), 31% (*p* < 0.01), and 51.3% (*p* < 0.01) in treatment groups with 0.2, 0.4, 0.8 μg/mL of EASs for 48 h, respectively (Figure 4B).

**Figure 5:**
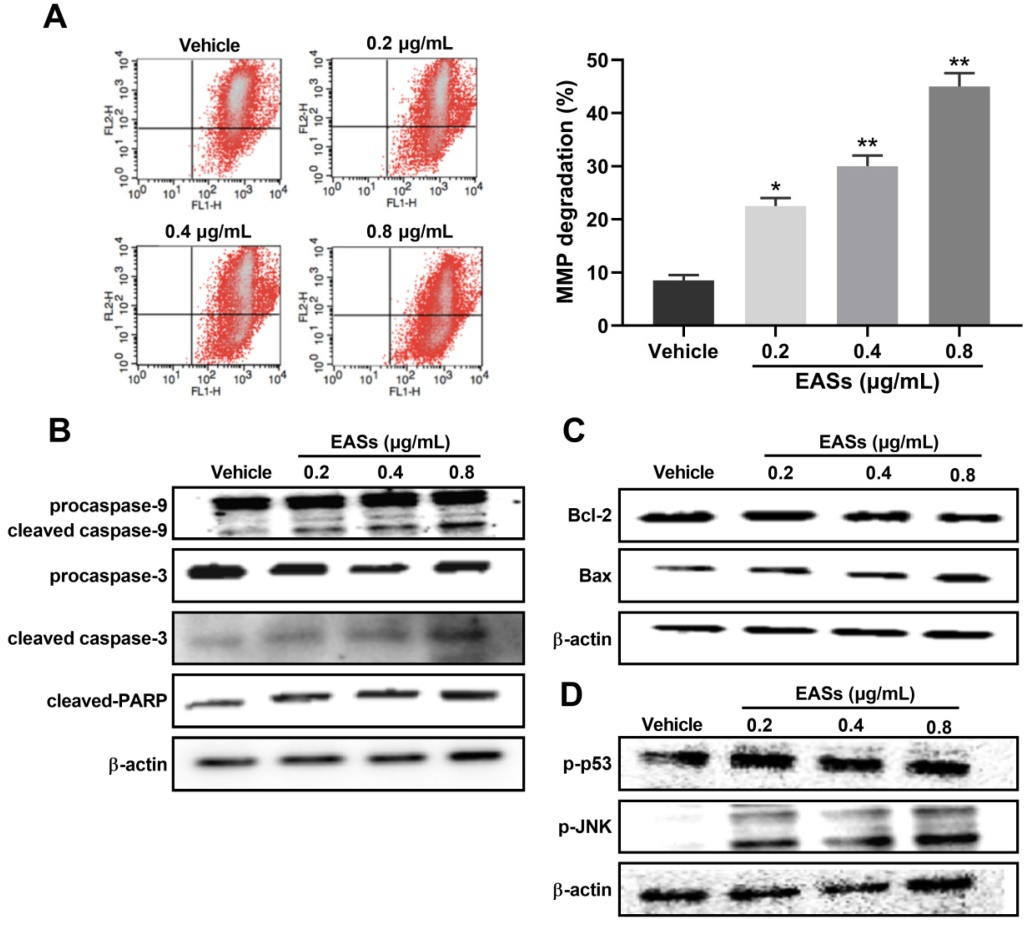
** Effect of EASs on MMP and apoptosis-related proteins.** (A) MMP changes in HepG2 cells by staining with JC-1 and evaluating on a flow cytometry induced by different concentrations (0.2, 0.4, and 0.8 µg/mL) of EASs for 48 h. Data were expressed as mean ± SD (*n* = 3). *p < 0.05, **p < 0.01, vs. vehicle. (B-D) The expressions of apoptosis-related proteins (procaspase-9, cleaved caspase-9, procaspase-3, cleaved caspase-3, cleaved-PARP, Bax, Bcl-2, Bid, p-p53, and p-JNK) in HepG2 cells treated with EASs for 48 h were analyzed using western blot.

For investigating the underlying mechanisms of apoptosis caused by EASs, analysis of MMP and apoptosis-related proteins were executed (Figure 5). Data on flow cytometry showed that EASs exerted a remarkable and concentration-dependent effect on declining the MMP, demonstrating that they induced apoptosis through intrinsic pathways (Figure 5A). As compared to the vehicle group, the percentage of cells with depolarized mitochondria augmented by 2.7- (*p* < 0.05), 3.6- (*p* < 0.01) and 5.4-fold (*p* < 0.01) after incubation with 0.2, 0.4 and 0.8 μg/mL of EASs, respectively. In addition, the expressions of apoptosis-related proteins in cells were determined using western blot. EASs concentration-dependently upregulated the expressions of cleaved caspase-3, cleaved caspase-9, cleaved-PARP, and Bax, while downregulating the expressions of procaspase-3, procaspase-9, and Bcl-2 (Figure 5B and C). At the same time, the phosphorylation levels of p53 and JNK in EASs-treated cells were higher than those in the vehicle group (Figure 5D). The above changes indicated the activation of the mitochondria pathway resulted from EASs, accounting for apoptosis of HepG2 cells.

### EASs resulted in ROS generation related to apoptosis in HepG2 cells

**Figure 6:**
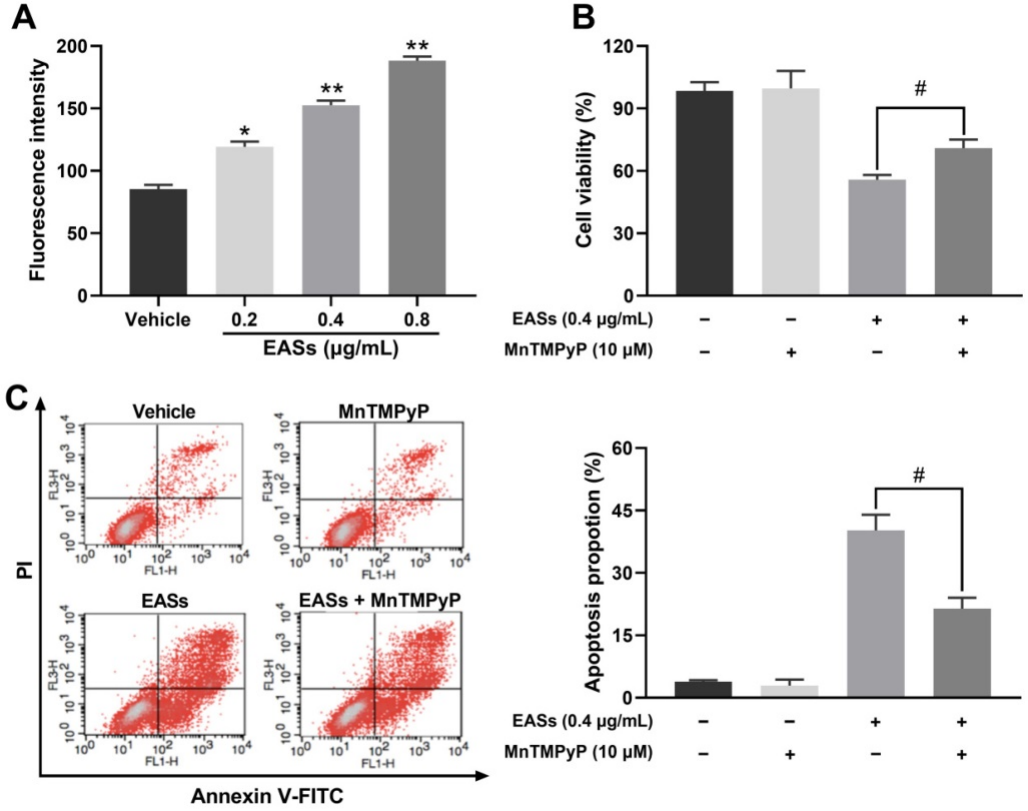
** EASs resulted in ROS generation related to apoptosis in HepG2 cells.** (A) Intracellular ROS level in HepG2 cells caused by different concentrations (0.2, 0.4, and 0.8 µg/mL) of EASs for 48 h was measured on a flow cytometry after stained with DCFH-DA. (B) HepG2 cells after EASs treatment (0.4 µg/mL) for 48 h with or without pre-treatment of MnTMPyP (10 µM). Cell viability was determined using MTT assay. (C) Apoptotic rate of HepG2 cells resulted from EASs (0.4 µg/mL) for 48 h in presence or absence of MnTMPyP (10 µM) was assessed by a flow cytometry. Data were expressed as mean ± SD (*n* = 3). *p < 0.05, **p < 0.01, vs. vehicle; ^#^*p* < 0.05, vs. treatment group without MnTMPyP.

As displayed in Figure 6A, the intracellular ROS level in HepG2 cells increased by 39.5% (*p* < 0.05), 78.5% (*p* < 0.01), and 120.5% (*p* < 0.01) respectively after exposure to various concentrations of EASs in comparison to the vehicle group. MnTMPyP was used to explore if ROS involves in causing apoptosis. No prominent differences in cell viability and apoptotic proportion of HepG2 cells cultured with or without MnTMPyP were found, manifesting a non-toxicity impact of 10 μM of MnTMPyP on HepG2 cells. It was observed that the addition of MnTMPyP notably increased the EASs-induced cell viability from 55.8% to 70.9% (Figure 6B, *p* < 0.05). The apoptotic activity was reversed via pretreatment with MnTMPyP which decreased the apoptotic rate from 40.2% induced by EASs to 21.4% (Figure 6C, *p* < 0.05). The above results suggested that ROS played a key role in triggering the apoptosis caused by EASs.

### Toxicity of EASs* in vivo*

**Figure 7:**
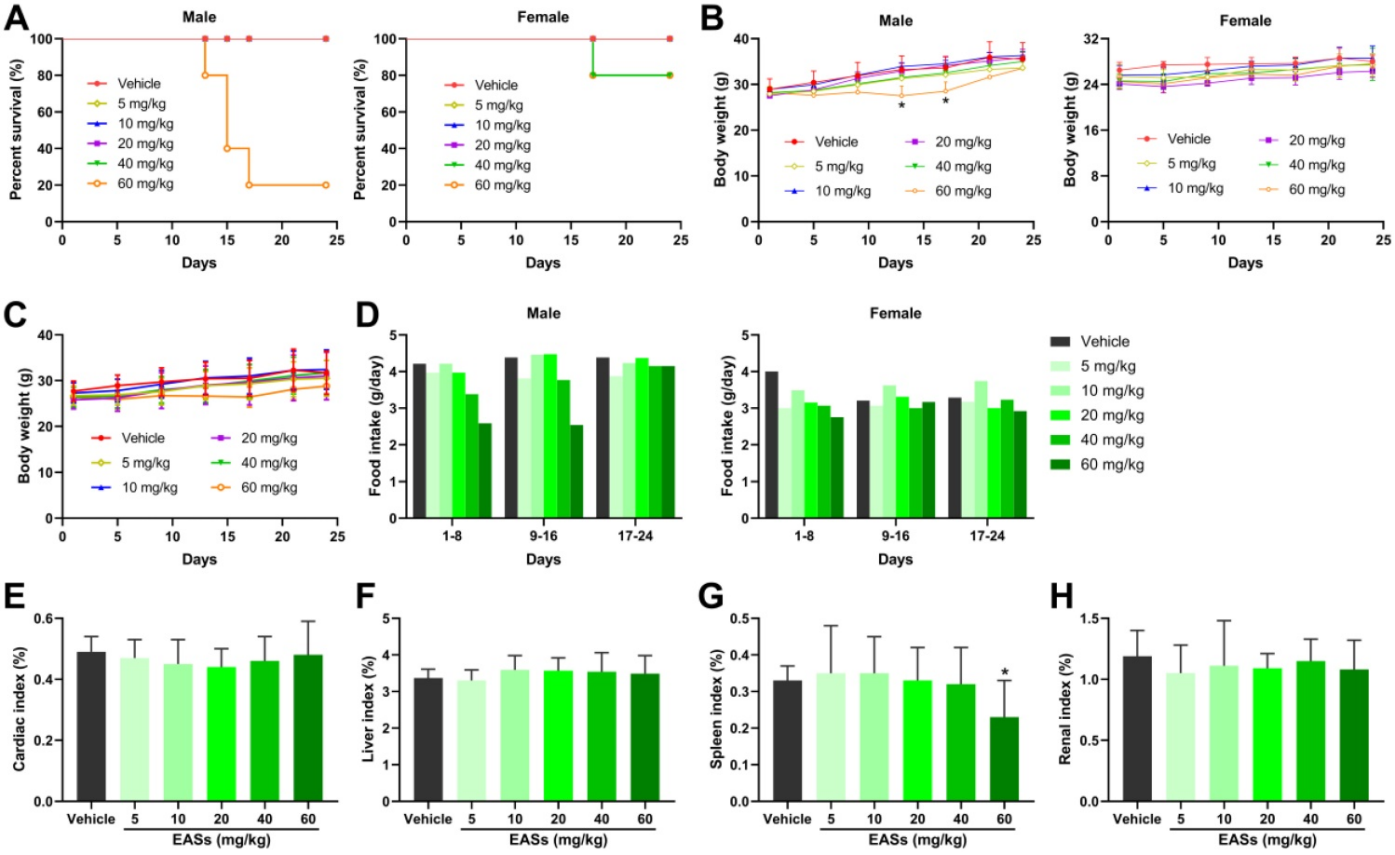
** Toxicity analysis of EASs *in vivo*.** (A) The survival curve of male and female mice respectively. KM mice were randomized into 6 groups (*n* = 10) containing 5 males and 5 females and intraperitoneally injected with EASs at doses of 5, 10, 20, 40, and 60 mg/kg or vehicle every day for 24 days. The survival rate was calculated every day. (B) Body weight curve of male and female mice respectively. Body weight was measured every 4 days. (C) Average body weight curve of mice in different groups. (D) Food intake of male and female mice respectively was measured every 8 days. (E-H) Cardiac index, liver index, spleen index, and renal index of mice in different groups were measured. Data were expressed as mean ± SD. *p < 0.05, vs. vehicle.

Prior to antitumor experiment* in vivo*, toxicity analysis of EASs was performed in mice and displayed in Figure 7. The survival rates in the male mice group after administration of the highest dose of EASs (60 mg/kg bw) reduced to 20%, whereas reduced to 80% in the female mice group treated with two higher doses of EASs (40 and 60 mg/kg bw) (Figure 7A). EASs declined the food intake in female mice group in the first 8 days, but not affect the food intake for the rest of the day relative to the vehicle group (Figure 7D). In the male mice group, only treatment with 40 and 60 mg/kg bw of EASs reduced the food intake. In addition, there were no significant variations in body weight in total mice groups and the female mice group, while the body weight in the male mice group that were supplied with 60 mg/kg bw of EASs obviously decreased in comparison with the vehicle group (Figure 7B and C, *p* < 0.05). Except for the spleen index of mice treated with the highest dose of EASs (60 mg/kg bw) was lower than that of the vehicle group (*p* < 0.05), EASs made no distinct differences to the normal organs including heart, liver, spleen, and kidney (Figure 7E-H). These results denoted that EASs (at most 40 mg/kg bw) had no obvious toxic effects on mice.

### EASs suppressed the tumor growth in an animal model

**Figure 8:**
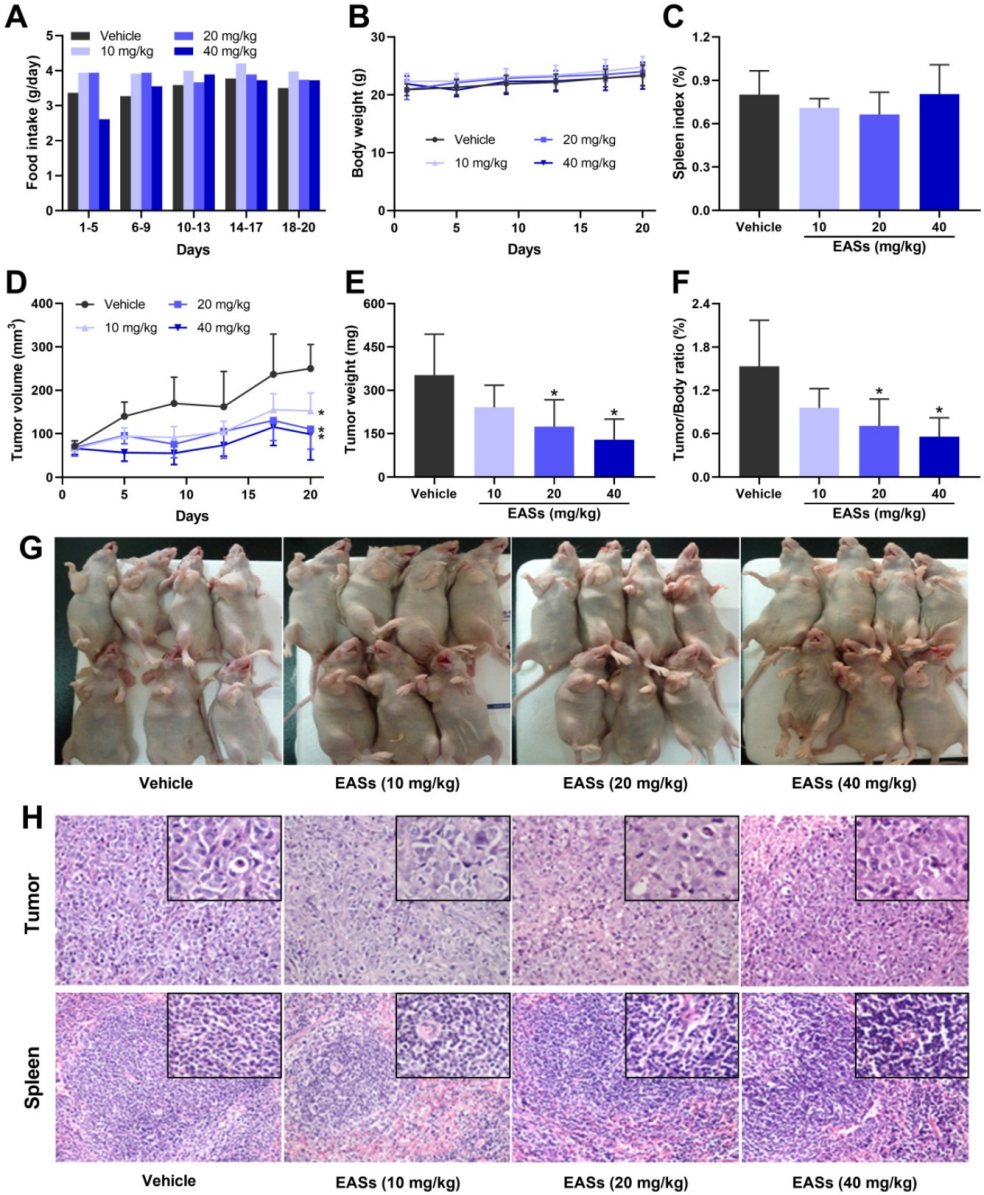
** EASs suppressed the tumor growth *in vivo*.** (A) Food intake of mice in different groups. HepG2 cells were implanted subcutaneously into the right axilla of BALB/c nude mice. Mice were randomly divided into 4 groups (*n* = 7) and treated with EASs (10, 20, and 40 mg/kg) or vehicle by intraperitoneal injection daily for 20 days. Food intake was measured every 5 days (B) Body weight curve of mice in different groups. Body weight was measured every 4 days. (C) Spleen index of mice in different groups was measured. (D) Tumor growth curve of mice in different groups. Tumor volume was calculated every 4 days. (E) Tumor weight in different groups was measured. (F) Tumor/Body ratio of mice in different groups was calculated. (G) An image of mice bearing tumor xenograft in different groups. (H) Representative images of H&E staining in tumor and spleen tissues (×200 magnification, the upper rectangles show the detail of each panel at ×400 magnification). Data were expressed as mean ± SD. *p < 0.05, vs. vehicle.

Three doses of EASs (10, 20, and 40 mg/kg bw) were applied to the antitumor experiment *in vivo* based on its toxicity. To assess the anti-cancer effect of EASs *in vivo*, a xenograft mouse model was established by injecting subcutaneously HepG2 cells to the right flank of nude mice and then the mice were given different doses of EASs for 20 days. During the first 5 days, the food intake of mice raised with 60 mg/kg bw of EASs was declined compared with that of the vehicle group (Figure 8A). Therefore, in the high-dose group (40 mg/kg bw), instead of administration every day, the mice were given EASs every other day after drug withdrawal for 5 days. Afterward, the food intake appeared no obvious changes among the four groups. Likewise, EASs had no evident impact on body weight and spleen index (Figure 8B and C). No apparent pathological alterations in sections of spleen were observed between treatment and vehicle groups (Figure 8H).

Moreover, EASs concentration-dependently decelerated the growth of tumor volume (Figure 8D and G). Consistent with the changing trend of tumor volume, the tumor weight of mice after supplementation with EASs was lighter than that of the vehicle group with a dose-dependent relation, leading to a decrease in tumor/body ratio (Figure 8E and F). Tumor/body ratio is one of the commonly-used indexes for evaluating the anti-tumor effects. Obviously, the tumor/body ratio decreased by 53.8% and 63.5% after treatment with 40 and 60 mg/kg bw of EASs respectively (*p* < 0.05). The results of H&E staining of tumor tissues revealed that fewer mitotic counts were found in the EASs-treated groups than those of the vehicle group, thereby inhibiting tumor growth (Figure 8H).

## Discussion

Owing to the adverse effects and toxicity of chemotherapeutic agents that are one of the main cancer treatments, natural products with profiles of natural security and low or non-toxicity are used as anti-cancer drugs more and more widely 25. Oats, a vital staple food for humans, are rich in numerous nutrients which are relevant to an abated risk of various types of cancer 26-28. Fermentation has been proved to improve nutritional features of oats through increasing the contents of total phenolics, flavonoids, and phenolic acids, and degrading antinutritional compounds like phytic acid, thereby enhancing the antioxidant and pancreatic lipase inhibitory activities 9, 15. In sight of these properties, fermented oats could serve as an alternative therapeutic approach against cancer. EASs from ethanol extracts of fermented oats by* Rhizopus oryzae* 3.2751 had the highest polyphenols content. Hence, the purpose of this work is to assess the anti-cancer activities of EASs rich in polyphenols from fermented oats.

Although the ability to kill cancer cells, current problems faced by chemotherapy and other anti-cancer drugs are able to kill normal cells around the cancer cells 29. Therefore, low cytotoxicity towards normal cells is critical for the development of anti-cancer therapy. Four common human cancer cells (MDA-MB-231, HepG2, HT-29, and HeLa cells) and two normal cells (3T3-L1 and ARPE-19 cells) were used to investigate the impact of EASs on cancer and normal cells. In our study, MTT assay indicated that EASs concentration-dependently suppressed the cell proliferation of four human cancer cells, while exhibiting weak cytotoxicity to normal cells (Figure 2A). Moreover, the colony formation was markedly decreased after EASs treatment with the increasing concentrations (Figure 2B). Additionally, EASs (less than the dose of 40 mg/kg bw) remarkably alleviated the growth of tumor volume and weight in tumor-bearing nude mice in a concentration-dependent manner with no significant organ-related and systemic toxicities (Figure 7 and 8). The evident anti-cancer activities *in vitro* and* in vivo* supported our hypothesis that EASs had the potential to be cancer drug candidates. Liver cancer has been the fourth predominant reason of cancer-related death around the world in 2018 and hepatocellular carcinoma is a major type of liver cancer 1, 30. Among four kinds of cancer cells studied, HepG2, HT-29, and HeLa cells had approximate sensitivity to EASs and showed more sensitive than MDA-MB-231cells. Given that, to uncover the deep mechanistic insights into the anti-proliferation effect of EASs on cancer cells, we firstly used only HepG2 cells (a variety of human hepatocellular carcinoma cells) in the further experiment.

Cell cycle progression is thought to have relevance to tumourigenesis and cell cycle disturbances are recognized to be a crucial tactic to eliminate cancer cells 31, 32. Several studies reported that natural products could inhibit the tumor growth at any phase of the cell cycle in different cancer cells, arousing disruption of the cell cycle. For instance, marmesin induced arrest in the G2/M phase of cell cycle in U937 cells with dose dependent 25. The rate of HeLa and Caski cells in G0/G1 phase was elevated after exposed to ferulic acid and the percentage of S phase cells was declined, implying a G0/G1 phase arrest 33. Treatment of HeLa cells with Lupeol brought about a prohibition in the S phase 34. The water-soluble fraction of *Kalanchoe tubiflora* led to A549 cell-cycle arrest primarily in G2/M phase and slightly in the G0/G1 phase 35. In our experiment, EASs exerted a dramatic accumulation in HepG2 cells in the G2/M phase with a concomitant decrease in the number of G0/G1 phase cells in the first 24 h (Figure 3A). Furthermore, a notable increase in the S and G2/M population of cells, accompanied by a reduction in the percentage of cells in the G0/G1 phase in the next 24 h (Figure 3B). Thus, EASs blocked the S and G2/M phase in HepG2 cells. A noticeable augment in the number of HepG2 cells in the sub-G1 phase was found (Figure 3), indicative of the apoptosis induction by EASs treatment. This was in coincidence with the literature that isorhynchophylline upregulated the population of HepG2 cells in the sub-G1 phase 36. The hallmark of G2/M and S phase arrest could be associated with the changes in cell cycle-related proteins, such as cdc25C, cdc2, p27, p21, cyclin, etc. 34, 37-39.

Apoptosis, a type of programmed cell death, is another resultful approach in a restriction of the proliferation of cancer cells 40. A number of researches suggested that one of the mechanisms of natural products on the tumor growth depression was realized either by resulting in apoptosis or arresting the cell cycle or a combination of both apoptosis and cell cycle interruption 28, 33, 41. Flow cytometry detection provides evidence that HepG2 cells incubated with EASs committed to apoptosis in a dose-dependent manner (Figure 4). It is well accepted that apoptosis occurs mainly via extrinsic or death receptor-mediated pathway and intrinsic or mitochondria-mediated pathway 42. In the extrinsic pathway, death receptors were bound with corresponding ligands when receiving extracellular signals, causing the formation of death-inducing signaling complex, activation of caspase-8 and caspase-3, and finally appearance of apoptosis 43. In other words, when cells suffered from the internal stimuli, the mitochondrial permeability transition pore opened, followed by the depolarization of MMP and the release of pro-apoptosis effectors to cytosol, such as cytochrome c (Cyt c). Cyt c could then lead to the activate of downstream caspase cascade through caspase-9 and caspase-3 which cleaved nuclear and cytoskeletal proteins, such as PARP 43, 44. Caspases usually exist in the form of inactive zymogens (procaspase) and go through autolytic cleavage to become active when provoked by upstream substances 45. It is thought that the modulation of mitochondrial membrane permeability involved in the members of Bcl-2 family proteins, including pro-apoptosis proteins (Bax, Bid, Bak, etc.) and anti-apoptosis proteins (Bcl-2, Bcl-xL, etc.) 43. During the development of apoptosis, Bcl-2 localized in the mitochondrial outer membrane to maintain the stability of mitochondrial membrane and suppress the release of Cyt c, while Bax translocated from cytosol to mitochondrial outer membrane, formed oligomers and facilitated the release of Cyt c. The Bcl-2/Bax ratio was generally considered as an indicator to evaluate the progression of apoptosis 46. The current study showed that treatment with EASs manifested a concentration-dependent loss of MMP in HepG2 cells (Figure 5A). Western blot analysis demonstrated that the expressions of Bcl-2, procaspase-9, and procaspase-3 were reduced and the expressions of Bax, cleaved caspase-9, cleaved caspase-3, and cleaved PARP rose in HepG2 cells exposed to EASs, rousing a decrease of Bcl-2/Bax ratio (Figure 5B and C). These findings indicated that EASs treatment probably activated a mitochondria-dependent apoptotic pathway.

p53 is regarded as a regulator of cell cycle and apoptosis progression by activating the downstream factors (p21, Bax) 47. Recent research pointed out that deoxyelephantopin regulated the apoptosis and cell cycle arrest in HCT 116 and KB cells directly by p53 which enhanced the expressions of p21, Bax, and p53 48. Meanwhile, an increase in phosphorylated p53 denoted that EASs could modulate p53-mediated signal transduction to pose the cell cycle arrest and apoptosis in HepG2 cells (Figure 5D).

ROS, produced by oxygen metabolism, acted as a vital role in the control of biological processes, including cell growth, apoptosis, inflammation, damage of cell membranes and DNA, and homeostasis 49. JNK is a major member of MAPK family proteins and correlated to proliferation, apoptosis, differentiation, and inflammation 50. Several studies stated that excessive ROS production caused by cellular stimulants could trigger the JNK signal pathways and eventually prevent cell growth 51, 52. We showed that EASs exhibited a significant and concentration-dependent augment in intracellular ROS generation in HepG2 cells (Figure 6A). In addition, MnTMPyP, a ROS inhibitor, obviously improved the cell viability and attenuated the apoptosis in EASs-treated HepG2 cells (Figure 6B and C). EASs promoted the phosphorylation of JNK dose-dependently, which induced the activation of JNK in HepG2 cells (Figure 5D). Wherefore, EASs led to mitochondria-dependent apoptotic signals to HepG2 cells likely via regulating the ROS/JNK signaling pathway.

## Conclusions

**Figure 9:**
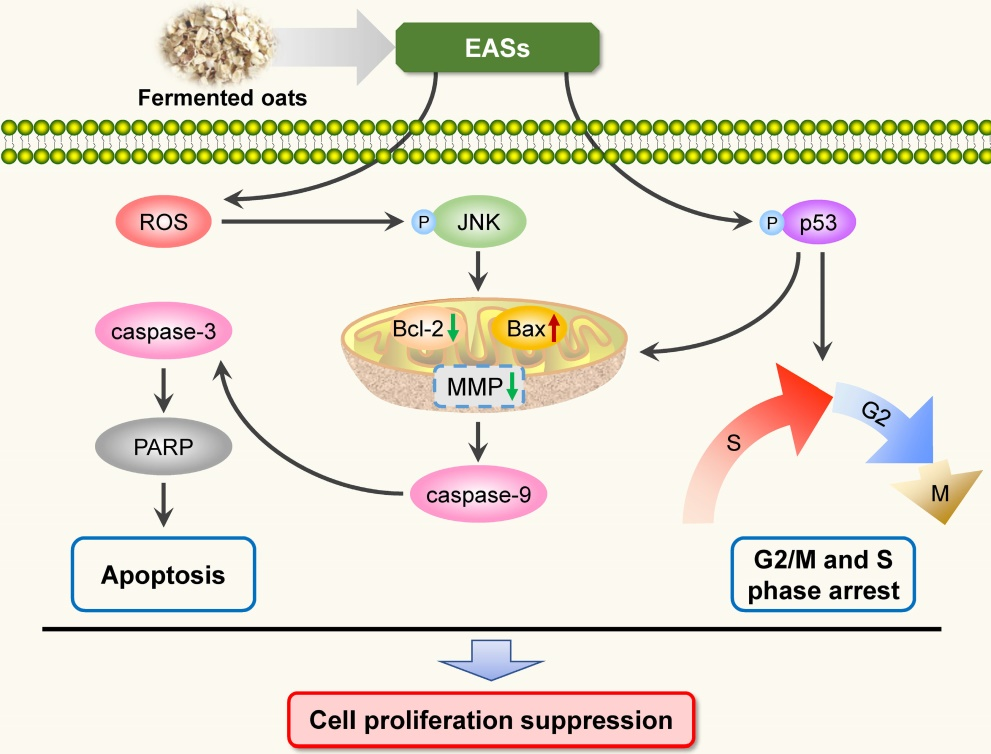
Schematic illustration of the pathways of EASs-induced cell arrest and apoptosis in HepG2 cells.

To sum up, the present study investigated the anti-carcinogenic activities of EASs from ethanol extracts of fermented oats by *Rhizopus oryzae* 3.2751 and its potential mechanisms of action. EASs suppressed the growth of four types of cancer cell lines (MDA-MB-231, HepG2, HT-29, and HeLa cells) with various IC_50_ values and exerted low cytotoxicity to normal cells (3T3-L1 and ARPE-19 cells). The number of colonies in HepG2 cells was markedly reduced when exposed to EASs. We demonstrated the ability of EASs to inhibit the proliferation of HepG2 cells via cell cycle arrest in G2/M and S phases and causing apoptosis (Figure 9). Initially, treatment with EASs to HepG2 cells enhanced the ROS generation and the phosphorylation of JNK and p53 in HepG2 cells. Then, EASs resulted in the mitochondria-dependent apoptotic pathway through the promotion of pro-apoptotic protein (Bax) and depression of anti-apoptotic protein (Bcl-2). Thereafter, caspase-9 was activated following by the activation of caspase-3, indicative of apoptosis. Furthermore, our data revealed that EASs (less than 40 mg/kg bw) alleviated the tumor growth *in vivo* without an apparent effect on food intake, body weight, and main organs (Figure 7 and 9). Thus, these results suggested that polyphenol-enriched composition from fermented oats might become a potential, novel and safer strategy for the future development as an efficacious agent against liver cancer. The further analysis involving in the specific bioactive components identified from EASs and effect of EASs on other forms of cancer will be executed.

## Abbreviations

EASs: ethyl acetate subfractions
DMSO: dimethyl sulfoxide
PI: propidium iodide
MTT: 3-(4,5-dimethylthiazol-2-yl)-2,5-diphenyltetrazolium bromide
RNase: ribonuclease
DCFH-DA: 2',7'-dichlorofluorescin diacetate
MnTMPyP: manganese (III) tetrakis-(1-methyl-4-pyridyl) porphyrin
MMP: mitochondrial membrane potential
JC-1: 5,5',6,6'-tetrachloro-1,1',3,3'-tetraethyl-benzimidazolylcarbocyanine iodide
PARP: poly-ADP-ribose polymerase
JNK: c-Jun N-terminal kinase
ROS: reactive oxygen species
H&E: hematoxylin and eosin
Cyt c: cytochrome c
